# Effects of somatic alterations at pathway level are more mechanism‐explanatory and clinically applicable to quantity of liver metastases of colorectal cancer

**DOI:** 10.1002/cam4.2368

**Published:** 2019-06-20

**Authors:** Zhong‐guo Zhang, Fei Ma, Shuang Zhao, Xiaoyu Yang, Fang Liu, Chenghai Xue, Liren Liu, Jin Gu, Haozhe Piao

**Affiliations:** ^1^ Large‐scale Data Analysis Center of Cancer Precision Medicine Cancer Hospital of Chinese Medical University, Liaoning Provincial Cancer Institute and Hospital Shenyang China; ^2^ Wankangyuan Tianjin Gene Technology, Inc Tianjin China; ^3^ Department of Gastrointestinal Cancer Biology National Clinical Research Center of Cancer, Tianjin Medical University Cancer Institute and Hospitasl Tianjin China; ^4^ MOE Key Laboratory of Bioinformatics, Beijing National Research Center for Information Science and Technology, Department of Automation Tsinghua University Beijing China

**Keywords:** algorithm, cancer pathways, liver metastases lesions, somatic alterations, survival

## Abstract

**Background:**

The quantity of metastases lesions is an important reference when it comes to making a more informed treatment decision for patients with colorectal cancer liver metastases. However, the molecular alterations in patients with different numbers of lesions have not been systematically studied.

**Methods:**

We investigated somatic alterations and microsatellite instability (MSI) of liver metastases from patients with single, multiple or diffuse metastasis lesions. A new algorithm “Pathway Damage Score” was developed to comprehensively assess the functional impact of somatic alterations at the pathway level. Pathogenic pathways of different metastasis were identified and their prognosis effects were evaluated. Furthermore, the subnetworks and affected phenotypes of the altered genes in each pathogenic pathway were analyzed.

**Results:**

Somatic alterations and altered genes occurred sporadically as well as in MSI state in different metastasis types, although MSS patients had more metastatic lesions than that of the MSI patients. Every metastasis group has their own pathogenic pathways and damaged “Cargo recognition for clathrin‐mediated endocytosis” is significantly associated with poor prognosis (*P* < 0.001). Further pathway subnetwork analysis showed that except conventional drivers, other genes could also contribute to metastasis formation.

**Conclusions:**

Progression of liver metastasis could be driven by the coefficient of all altered genes belonging to the pathways. Thus, compared to somatic alterations and genes, pathway level analysis is more reasonable for functional interpretations of molecular alterations in clinical samples.

## 1 INTRODUCTION

Colorectal cancer (CRC) has one of most high mortality rate cancers in the world. CRC liver metastasis (CRCLM) is the most common type of CRC metastasis.^1^ There is growing incidence of CRC with liver metastasis among younger patients in recent years.^2^ In population‐based materials, the 10‐year OS ranges from 4.6% to 15.1% depending on the number of liver metastases and surgical resection.^3^ The number of liver metastases which is closely related to liver function after treatment is an important reference for medical professionals to have an operation.^4^ However, these clinical factors alone are not sufficient enough to predict the clinical outcomes.

Molecular characteristics are very useful for the prognosis of CRC.^5^ Genetic alterations caused cancer occurrence and distant metastasis had been viewed in some studies.^6^, ^7^, ^8^, ^9^ A study using large‐scale panel sequencing (about 400 cancer genes) of metastatic CRCs with both primary tumors and metastases have defined some genetic alterations closely related to clinical features.^10^ A previous study found that activation of RAS by a small GTPase Ras signal can lead to a series of downstream phosphorylation events in the RAF‐MEK‐ERK cascade and induces cell proliferation and migration in CRCLMs.^11^ Jelena Urosevic found that activation of *ERK2* not *ERK1* can provide colon cancer cells with the ability to seed and colonize the liver.^12^ Yan also identified a CRCLM related lncRNA named *LUCAT1* by impairing cancer cell invasion.^13^ However, most of these studies focused on a small number of genes or pathways, which did not get the comprehensive effect of all coding alterations to find the molecular features related to important and subdivided clinical factors such as the quantity of metastases lesions in the CRCLM.

In this study, we investigated the genome‐wide exonic variants and microsatellite characteristics of metastatic tumor in CRCLM patients with different number of metastatic lesions using whole exome sequencing. To study the holistic effect of all coding alterations rather than only on traditional cancer genes, a new algorithm named “Pathway Damage Score” (PDS) which could evaluate the accumulative effect of all alterations on tumorigenesis was developed. Additionally, alteration effects on pathway level calculated using algorithm based on all intragene alterations were used to explore the molecular dynamics and indicators for clinical features.

## 2 MATERIALS AND METHODS

### 2.1 Patient cohorts and sample selection

A total of 15 patients diagnosed with single lesion (defined as type I) or multiple lesions (2‐15 metastases, defined as type II) or diffused lesions (more than 15 metastases, defined as type III) of CRCLM who had received surgeries between 2016 and 2017 without prior treatment including chemotherapy or radiotherapy at LIAO NING CANCER HOSPITAL & INSTITUTE were enrolled (Figure S1). TNM stage of patients ranged from III to IV according to AJCC 7th edition.^14^ All tumor samples and matched adjacent normal samples were collected freshly during surgery and stored in liquid nitrogen immediately. All tumor samples that had >80% tumor content after microdissection, as determined by the routine hematoxylin and eosin stain done by two independent pathologists, were included. Each collected sample weighted at least 100 mg and was typically under 200 mg.

Collection and use of all specimens in this study were approved by the Large‐scale Data Analysis Center of Cancer Precision Medicine, Liaoning Provincial Cancer Hospital & Institute (Cancer Hospital of China Medical University). The study protocol was approved by the ethics committee of the Liaoning Provincial Cancer Hospital & Institute (Cancer Hospital of China Medical University) and was conducted in accordance with the Declaration of Helsinki. Informed consent was obtained from all participants.

### 2.2 Exome capture and sequencing

Genomic DNA was extracted with QIAamp DNeasy Blood & Tissue Kit (Qiagen) from frozen tissue samples. Up to 3 µg of genomic DNA was fragmented into a base‐pair peak of 250‐300 bp fragments. Adaptor‐ligated templates were purified using Agencourt AMPure XP beads, and fragments with an insert size of ~250 bp were isolated. In‐solution exome capture was carried out using the SureSelect Human All Exon V6 kit (60 Mb) (Agilent Technologies). Paired‐end sequencing, resulting in sequences of 150 bases from each end of the fragments, was performed on the HiSeq4000 platform following the manufacturer's instructions (Figure S1).

### 2.3 Sequencing data analysis

Low quality reads and adapter sequence were removed using Trimmomatic^15^ and data with Q30 > 93% were aligned to the hg19 human reference genome using Burrows‐Wheeler Aligner.^16^ Samples with mapped reads <150X were additionally sequenced to reach 150X. Overall an average mappable read coverage of 200X was achieved (Table S1). BAM files were then sorted and removed duplicate reads using the SAMtools softwore,^17^ base quality scores of reads were re‐calibrated by the BaseRecalibrator subroutine of Genome Analysis Toolkit (GATK),^18^ and somatic SNV/Indels in the exom region were identified by Mutect2.^19^ The SNV/Indels marked “PASS” by Mutect2 were annotated using SnpEff,^20^ Variant Effect Predictor^21^ and Loss‐Of‐Function Transcript Effect Estimator.^22^ We filtered out known polymorphisms variants documented in (a) the dbSNP138; (b) the 1000 Genomes Project and (c) Exome Aggregation Consortium. Somatic copy‐number alternations were identified for each gene by comparing the normalized average per‐base coverage rate in a tumor sample to the normalized average per‐base coverage rate in the matched normal sample from the same individual.^23^ We selected fold change thresholds ≥ 3.0 and < 0.25 for calling amplifications and homozygous deletions. We used Meerkat^24^ to characterize the spectrum of SVs at base‐pair resolution in the tumor sample and matched normal sample. Then, somatic SVs were defined as not only existent in tumor sample but also nonexistent in matched normal samples.

We used MSMuTect^25^ to detect somatic MS indels. We classified tumors samples as microsatellite stable (MSS, MS indel < 3), low microsatellite instability (MSI‐L, A‐motif indel ≥ 3 but < 10) and high microsatellite instability (MSI‐H, A‐motif indel ≥ 10).^25^ MS motifs such as C‐motif, AC‐motif and AG‐motif were also identified as well as their repeat number. To explore the clinical significance of MSI characteristics, we calculated linear relations between metastases number and MS indel number and different motif repeat number.

### 2.4 PDS algorithm and pathogenic pathways determination

We proposed that pathway abnormality is a key factor for tumorigenesis. The degree of pathway damage depends on the effects of alterations on genes and its interaction pattern in the pathway. The effects of an alteration on a transcript were determined by confidence of alteration detection (defined as MC), degree of dysfunction (defined as ME), and enhancement or restraint of gene function that can be interpreted as effect direction (defined as MED). So, the effect score of a alteration on a transcript (TMES) can be calculated as:

(1) TMES=MC×ME×MED

The valuation of MC, ME and MED can be learnt from supplementary methods.

A genomic alteration usually can affect several transcripts of a gene at the same time, but we did not know which transcripts were the major components in the cell. Therefore, we used the average TMES of all effected transcripts of a gene to score the effect of an alteration on the gene.

(2) $$\text{GDS}=1/m\sum_{k=1}^{m}{\text{TMES}}_{k}$$

where GDS is the effect score of an alteration on a gene, TMES*_k_* is the effect score of an alteration on transcript *k*, *k* = 1, 2, ..., *m*, *m* is the number of effected transcripts of the gene.

To evaluate the significance of a certain gene in a pathway, we calculated the number of interacted genes of this certain gene in this pathway to represent the gene's importance weight (GIW) in this pathway. The Pathway, gene‐gene interaction and gene‐pathway information were from the PathwayCommons database.^26^ So, for a pathway, its damage score (PDS) can be defined as

(3) $$\text{PDS}=\sum_{j=1}^{n}\left({\text{GIW}}_{j}\times {\text{GDS}}_{j}\right)$$

where GIW*_j_*_,_ *j* = 1, 2, …, *n* is the importance weight of the gene(*j*) in the pathway, GDS*_j_* is the gene effect score calculated from its somatic variants. Merging mathematic formulas (1), (2), (3), we can form an integrated calculation formula as follows:

(4) $$\text{PDS}=\sum_{j=1}^{n}\left({\text{GIW}}_{j}\times {\left[1/m\sum_{k=1}^{m}\left({\text{MC}}_{k}\times {\text{ME}}_{k}\times {\text{MED}}_{k}\right)\right]}_{j}\right)$$

**Figure 1:**
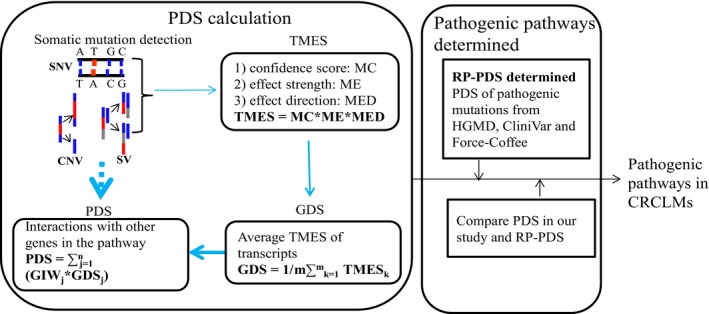
Schematic diagram of PDS algorithm. TMES: effect score of a alteration on a transcript; GDS is the damage score of an alteration on a gene, TMES*_k_* is the effect score of an alteration on transcript K, *k* = 1,2,..., *m*, *m* is the number of effected transcripts of a gene; GIW*_j_*_,_ *j* = 1,2, … *n* is the importance weight of gene(*j*) in the pathway; PDS is the damage score of pathway derived from all alterations of genes in this pathway (See methods).RP‐PDS were calculated with the PDS algorithm based on pathogenic alterations from HGMD, CliniVar and Force‐Coffee database. If the PDS of a pathway based on somatic alterations in our study was greater than RP‐ PDS, then this pathway was considered as the pathogenic pathway in our study.

We use formula (4) to calculate PDS of every pathway in every sample (Figure1).

To estimate what degree of PDS of pathways can be pathogenic for tumor progression, we use PDS calculated by known pathogenic alterations from cancer pathogenic database HGMD,^27^ CliniVar^28^ and Force‐Coffee^29^ as a reference PDS (RP‐PDS). If the PDS of a pathway in the sample was greater or equal to that in the RP‐PDS, then the pathway was defined as pathogenic in this sample.

### 2.5 Mutated subnetworks of pathogenic pathways estimation

For subnetworks, all mutated genes and their direct interacting genes which were involved in cancer related biology processes such as cell proliferation, migration, genomes instability, cell death, angiogenesis, immune response and inflammatory response, were defined as nodes and interactions defined as edges. The networks were plotted using SVG program.

### 2.6 Statistical analysis

The difference in quantity of metastasis lesions between MSS and MSI‐L samples were evaluated with the Wilcoxon paired test. We used somatic alterations (SNV and CNVs) and overall survival information (from pathologic diagnosis to last follow‐up) from 416 colon cancer patients registered in the project “The Cancer Genome Atlas” (TCGA) to evaluate the prognosis predictor of pathogenic pathways (samples without somatic alterations, and overall survival time equal 0 were excluded). Kaplan‐Meier survival function was calculated and compared with the log‐rank test. GraphPad Prism 5 software was used for all statistical analyses and *P* < 0.01 was considered significant.

## 3 RESULTS

### 3.1 Molecular alterations in liver metastasis of colon cancer

**Figure 2:**
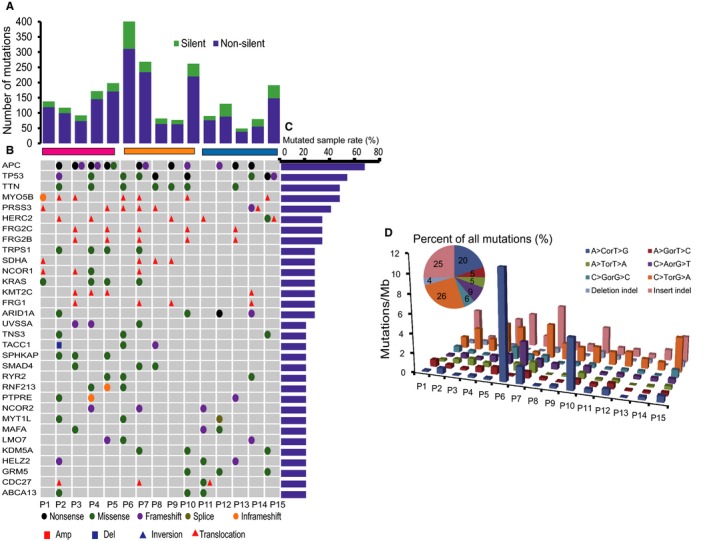
Genome‐wide somatic alterations landscape of CRCLMs. (A) Number and type of alterations. Nonsilent alterations consist of missense and nonsense substitutions, exomic indels and substitutions at splicing sites. (B) Landscape of somatic alterations of recurrent mutated genes (mutated in at least three samples) in samples of three metastasis types. Purple, orange and blue bar marketed type I, II and III respectively. Genes were arranged vertically by proportion of mutated samples. (C) Proportion of samples with somatic alterations that targeted each gene. Proportion was calculated as the percentage of individuals with nonsilent alterations for each gene. (D) “Lego” plots of alteration frequencies across 15 CRCLM samples for whole exon region. Base substitutions are divided into eight categories (six types of single base substitutions and two types of indels) to represent the eight possible base changes (each category represented by a different color). The inset pie chart indicates the distribution of all alterations for 15 CRCLMs mutated bases across the territory being evaluated

In total, 453 silent and 1904 non‐silent somatic mutations were identified, corresponding to 127 nonsilent mutations per tumor (range of 38‐311) or 2.54 (0.76‐6.22) per mega base (Table S2). Nonsilent alterations were 4.48‐fold more than silent alterations on average (range from 2.10 to 6.88) (Figure 2A). All these mutations were located on 1568 genes and affected 4531 different transcripts. There were also 28 genes with somatic amplification and 228 genes with somatic deletions (Figure S2). Additionally, 183 somatic translocations and two somatic inversions were also identified in patients (Table S2).

**Figure 3:**
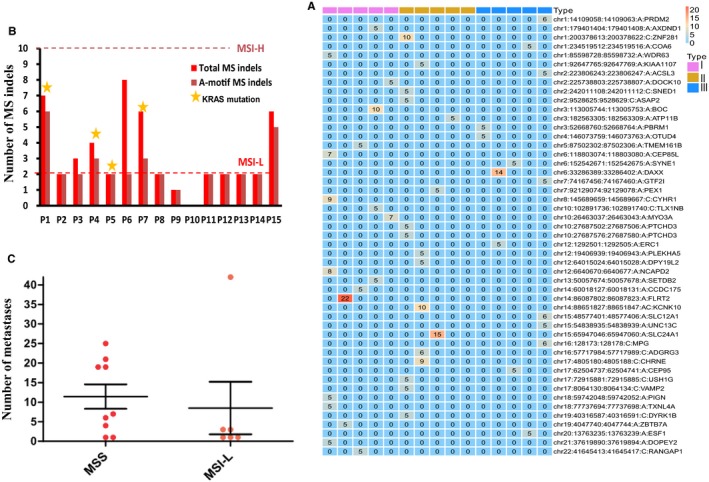
Somatic MSI and its clinical characteristics in CRCLMs. (A) MS indel characteristics in patients. MS indels were named in “chr:genome‐location (start:end):repeat‐motif:gene” format which indicates genome location, repeat motif and host gene of MS indel in rows, number in gridding represents times of repetition. Samples were arranged horizontally by metastasis number and grouped in type I, II and III. (B) The total number and the number of A‐motif MS indel classified MSS and MSI patients in our study. MSS and MSI were distinguished by total MS indel ≥ 3, MSI‐L and MSI‐H were distinguished by A‐motif indels ≥ 10. KRAS alterations were labeled to show its relation with MSI state. (C) Metastatic lesions in MSS and MSI patients

Genes altered nonsilently in more than three patients were regarded as recurrent. In our study, there were 32 recurrent altered genes, including conventional cancer genes such as *APC* (67%), *TP53* (53%) and *KRAS* (27%) (Figure 2C).The most common types of base conversion in the exomic region were C > T transition (26%) in eight alteration categories (Figure 2D), which is consistent with spontaneous cytosine deamination^30^ being a major mutagenic process in CRCLMs. Forty nine different somatic MS indels were identified in 48 genes and the A‐motif MSI was the major type in colon cancer (Figure 3A, S3). These results were consistent with previous studies.^24^, ^31^ All patients were MSS (60%) or MSI‐L (40%), consistent with the fact that MSI is more common among stage I or II (~20%) than in stage III (~12%)^32^ and stage IV CRC (~4%).^33^ KRAS alterations are more likely to be observed in MSI (50%) than MSS (11%) CRCs (Figure 3B), which is consistent with the description of these alterations as early key events that lead to intermediate adenomas within the pathogenesis of sporadic CRC.^34^ Additionally, the total MS indel and total repeat length of “A” motif were significantly negatively correlated to metastasis number in multiple metastasis CRCLMs (Figure S3). Although there were more metastases lesions in MSS patients than that in MSI‐L, patients with different types of metastases lesions could not be classified by MSI state (Figure 3C). Objectively, all these mutated genes, alterations and MSI features exhibit a sporadic pattern across different numbers of metastatic lesions.

### 3.2 Pathogenic pathways leading to formation of different quantity of metastasis lesions

**Figure 4:**
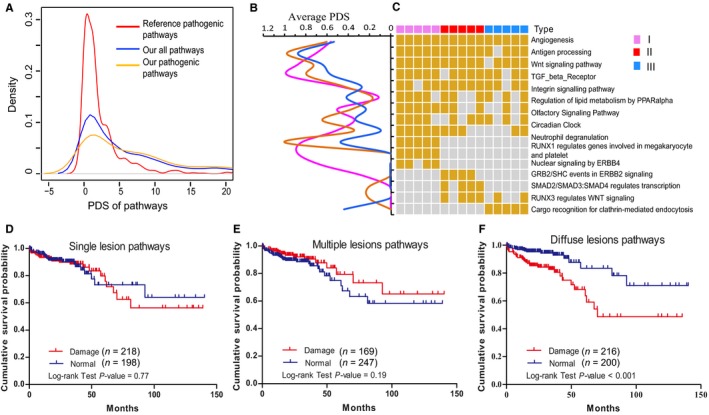
Pathogenic pathways identified by PDS algorithm in CRCLMs. (A) Distributions of PDS in reference pathogenic pathways, pathogenic pathways and all pathways detected in our study. (B) Trend lines of average damage score of pathogenic pathways in every different metastasis group, indicates that different pathways have different damage scores in different types of metastasis samples. Metastasis type I, II and III were represented by Purple, red and blue, respectively. (C) Heat map of key pathogenic pathways in the formation of liver metastases derived from primary tumor and different metastatic lesions. (D‐F) Kaplan‐Meier plot of overall survival and damage of group‐specific pathogenic pathways in TCGA colon cancer patients. Single lesion pathways included “RUNX1 regulates genes involved in megakaryocyte differentiation and platelet function,” “Nuclear signaling by ERBB4” and “GRB2/SHC events in ERBB2 signaling,” multiple lesions pathways included “SMAD2/SMAD3:SMAD4 heterotrimer regulates transcription” and “RUNX3 regulates WNT signaling,” diffuse lesions pathway included “Cargo recognition for clathrin‐mediated endocytosis.”

To determine the pathogenic damaged pathway, PDS calculated by known cancer pathogenic alterations were used to determine pathogenicity of damaged pathways in our study (see Methods). This helped us to investigate the pathogenic mechanism at a more systemic level of the pathway rather than at the gene level or the alteration level. Compared to RP‐PDS, Gaussian distribution of PDS of pathogenic pathways had a larger variance in our study. This suggests that both conventional and non‐conventional pathogenic alterations can be contributions of pathogenic PDSs. Additionally, PDS distribution of all pathways in our study revealed that nonpathogenic pathways carried lower PDS than pathogenic pathways (Figure 4A). This trend was also reflected in single patient's PDS distribution (Figure S5).

We investigated the pathogenic pathways related to quantity of metastatic lesions. Nine common pathogenic pathways were identified in CRCLMs. The angiogenesis pathway damaged pathogenically in all CRCLM patients indicated that blood vessels are the common or probably the only way for tumor cells to migrate from the colon to liver. Wnt, TGF beta receptor and antigen processing which are closely related to colon cancer progression and immune response were also identified as pathogenic in almost all patients. Integrin signaling and cell surface interactions in the vascular wall pathway which influence the interactions between cell surface receptors and the extracellular matrix (ECM) or the actin cytoskeleton were damaged in 87% (13/15) and 73% (11/15) patients, respectively. These results indicated that these pathways may maintain common phenotypes of tumor cells such as proliferation and colon‐liver metastases. Other common pathogenic pathways like the PPAR alpha‐regulated lipid metabolism could be involved in the tumor microenvironment (Figure 4B,4C).

Importantly, we identified special pathogenic pathways for different CRCLM groups using the PDS algorithm. Single lesion: “RUNX1 regulates genes involved in megakaryocyte differentiation and platelet function”, “Nuclear signaling by ERBB4” and “GRB2/SHC events in ERBB2 signaling”; multiple lesions: “SMAD2/SMAD3:SMAD4 heterotrimer regulates transcription and RUNX3 regulates WNT signaling”; diffused lesion: “Cargo recognition for clathrin‐mediated endocytosis” (Figure 4C). These pathways were likely to play important roles during metastasis^35^, ^36^, ^37^, ^38^ and could be used to classify CRCLMs with different metastatic lesions in clinical practice. Damaged “Cargo recognition for clathrin‐mediated endocytosis” pathway was significantly associated with poor overall survival in TCGA colon cancer patients, which indicated that this pathway could be a prognosis predictor for colon cancer and patients with diffuse liver metastasis were likely to survive only for a short period of time(Figure 4D‐F). Additionally, 239 pathogenic pathways clustered into four pathway groups sporadically across different CRCLMs (Figure S6). These pathways were supposed to inflict weak influence and could not be used as clinical markers in CRCLMs. Other nonpathogenic pathways usually had lower PDS and no clustering effect as sporicidal pathogenic pathways (Figure S7).

### 3.3 Mutated subnetwork of special pathogenic pathways of different type of CRCLMs

**Figure 5:**
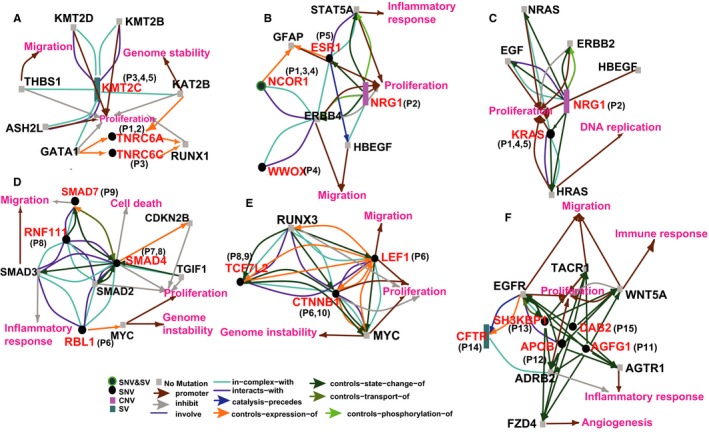
Subnetworks of special pathogenic pathways corresponded to different metastasis lesions. The key pathogenic pathways in single metastases were RUNX1 regulating genes involved in megakaryocyte differentiation and platelet function signaling (A), Nuclear signaling by ERBB4 (B) and GRB2/SHC events in ERBB2 signaling (C); The key pathogenic pathways in multiple metastases were SMAD2/SMAD3:SMAD4 heterotrimer regulating transcription (D) and RUNX3 regulating WNT signaling (E); The key pathogenic pathway in diffused metastases was Cargo recognition for clathrin‐mediated endocytosis (F). All correlations between genes and cell phenotypes (such as proliferation, migration, inflammatory response, and so on) were determined using Gene Ontology

To explore how specific pathogenic pathways affect cell phenotypes, and thus contribute to different metastases, the mutated subnetworks of these pathways were studied. KMT2C which mainly induces H3K4me1 at gene enhances, its coding gene mutated recurrently in cancers.^39^ In single lesion CRCLM, mutated KMT2C weakened cell migration, affected genome stability and cell proliferation through abnormal THBS1, KAT2B and proliferation inhibitors like GATA1 and KAT2B^40^, ^41^, ^42^ (Figure 5A). In the nuclear signaling by ERBB4 pathway, the promotion on cell proliferation and inhibition on cell migration were achieved by alterations on complex mate genes of ERBB4 in all single lesion CRCLMs^43^, ^44^ (Figure 5B). Moreover, mutated KRAS and NRG1 could affect cell proliferation and DNA replication through downstream targets like ERBB2, EGF and HRAS^45^ (Figure 5C). For multiple lesions CRCLM, SMAD family genes and their partners were dominant. Mutated SMAD4 could reduce the inhibition of SMAD2, CDKN2B, TGIF, and itself in cell proliferation. Inhibition of SMAD3 in inflammatory response could be weakened due to the abnormal complex formed by SMAD3 and mutated partners, and this could induce overreaction to the chemotherapy (Figure 4D). RBL1 and CTNNB1, TCF7L2 and LEF1 in RUNX3 regulating the WNT signaling pathway control the expression, state‐change and complex with MYC. Alterations of these genes could reduce cell proliferation as well as genome instability caused by MYC. In addition, alterations of CTNNB1‐TCF7L2‐LEF1 signaling cascade could lead to out of control of expression WNT signaling genes,^46^ and then affect cell proliferation directly and migration through RUNX3 (Figure 5D,E). In diffuse lesions CRCLM, all patients of this type carried the pathogenic pathway: Cargo recognition for clathrin‐mediated endocytosis. In this pathway, all mutated genes control the state of FZD4 directly or through ADRB2, and may thereby promote angiogenesis.^47^ EGFR and WNT5A were also important downstream targets of these genes to affect cell migration.^48^ Traditionally, EGFR promotes proliferation and migration mainly through conformational changes such as tyrosine phosphorylation.^49^ However, the pathogenicity of EGFR in our study may be due to the damage alterations in its interacting genes, and suggest another approach for the treatment of EGFR dysfunction. Alterations of the upstream genes of EGFR could attenuate the downstream immune response and cell migration, but its effect on cell proliferation could go either way, therefore no definitive conclusion could be drawn (Figure 5F).

## 4 DISCUSSION

Metastases lesions are an important reference in both surgery decision and prognosis prediction during CRCLM treatment. However, the molecular mechanism of formation and classified methods of single, multiple and diffuse CRCLM are still unknown. Alterations on gene level and MSI features were sporadic across patients, thus can not reveal mechanism of the formation of different quantity of metastasis. But when we transferred alteration affects onto the pathway level by PDS algorithm, the common pathogenic pathways for colon‐liver metastasis and lesion quantity‐special pathogenic pathways were successfully identified. So, evaluating the affect of alterations on cell phenotypes on pathway level is more accurate and systematic than that only on alteration itself or gene level.^50^, ^51^ The pathogenic pathways, especially type special pathogenic pathways provided important insights into molecular mechanisms. In general, for three types of CRCLM, the ability of cancer cell migration and genomic stability were gradually increased, while the immune response was gradually reduced which led to enhanced immune escape of cancer cell, but the trend of cancer cell proliferation appeared ambiguous (Table 1). Several nonclassical pathways impaired by non‐cancer altered genes also seem to play an important role in metastasis. Peroxisome proliferator‐activated receptor alpha is the major regulator of fatty acid oxidation in the liver. Abnormal regulation of lipid metabolism by PPAR‐alpha has been considered as the first alteration to cause HCC.^52^ In our study, it seems that damage of this pathway may also act as liver “soil” in formation of the metastasis of CRCLMs. The Olfactory Signaling pathway regulates migration and adhesion of muscle cells, control serotonin secretion by enterochromaffin cells and promote metastasis in pancreatic cancer.^53^, ^54^, ^55^ However, high frequency damage of this pathway in the CRCLM patients was identified firstly. As microsatellite instability is a key feature of colon cancer, we performed the PDS analysis between MSI‐L and MSS patients. Results indicated that the common damaged pathways of the two groups were not related to cancer progression directly as well as MSS special damaged pathways, while the MSI‐L special damaged pathways such as the “Formation of TC − NER Pre − Incision Complex” and “Nucleotide Excision Repair” were all related to the DNA repair system (Figure S8A). We also performed the pathway analysis between patients with the primary site on the left bowel and right bowel, and did not find the special damaged pathways related to either of them (Figure S8B).

**Table 1:** Effects of pathogenic pathways on cell phenotypes

CRCLMs	Special pathway	Phenotype				
		proliferation	migration	Inflammation response	Immune response	Genome instability
Single lesion	RUNX1 regulates genes involved in megakaryocyte differentiation and platelet function	P/I	I	NE	NE	P
	Nuclear signaling by ERBB4	P/I	I	I	NE	NE
	GRB2/SHC events in ERBB2 signaling	P	NE	NE	NE	NE
Multiple lesions	SMAD2/SMAD3:SMAD4 heterotrimer regulates transcription	P/I	P/I	P	NE	I
	RUNX3 regulates WNT signaling	P/I	P	NE	NE	I
Diffuse lesions	Cargo recognition for clathrin‐mediated endocytosis	P	P	P/I	I	NE

Abbreviations: P, promote; I, inhibit; NE, no effect.

Treatment benefits could also be obtained from assessing alteration effect on the pathways. On the one hand, type special pathogenic pathways are potential candidate molecular markers for predicting quantity of metastasis lesion formation. Damaged “Cargo recognition for clathrin‐mediated endocytosis” pathway can predict poor prognosis. Additionally, relatively smaller gene sets involved in particular pathways make our method more economical and stabilized in clinical application, which encourages us to develop it in large‐scale samples studies in the future. On the other hand, pathogenic pathways have some implications for therapeutic process. For example, “Clathrin‐mediated endocytosis” is important for endocytosis in the eukaryote cell. It can regulate cell signaling transduction by quickly removing or reducing tyrosine kinase receptor and G protein‐coupled receptors, as well as the activity of synapses and of transporters,^56^ thus slowing down the tumor progression. In our study, damaged Cargo recognition is likely to promote the intenity of cancer signaling like EGF and Wnt, and then resulted in overflow of tumor spreading. Therefore, against Clathrin‐mediated endocytosis may be a new direction of therapy for diffuse lesions CRCLMs. However, the results from calculations of reasonable algorithm in our study are still insufficient for treatment guidance of the CRCLM; functional experiments of them have to be carried out before clinical applications.

Although more accurate and comprehensive understanding of the effects of tumor alteration are acquired at the pathway level, tumor cell phenotypes were usually determined with various genes and pathways.^9^ For example, there were three pathogenic pathways that specially occurred in single lesion CRCLMs, the priority of these pathways in phenotype determination could not be recognized accurately in automatic mode with our PDS algorithm. This result suggests room for further development of the PDS algorithm in its clinical applications.

## CONFLICTS OF INTEREST

Xue, Zhao, and Ma are employees of Wankangyuan Tianjin Gene Technology, Inc No other disclosures were reported.

## AUTHOR CONTRIBUTIONS

ZZ and XY performed the experiments. FM and SZ worked on bioinformatics analysis. ZZ and FM wrote the manuscript with the help from all the authors. H. P., JG and LL conceived the experiment. HP, ZZ and JG supervised the project. All authors read and approved the final manuscript.
